# Localization of Short-Chain Polyphosphate Enhances its Ability to Clot Flowing Blood Plasma

**DOI:** 10.1038/srep42119

**Published:** 2017-02-10

**Authors:** Ju Hun Yeon, Nima Mazinani, Travis S. Schlappi, Karen Y. T. Chan, James R. Baylis, Stephanie A. Smith, Alexander J. Donovan, Damien Kudela, Galen D. Stucky, Ying Liu, James H. Morrissey, Christian J. Kastrup

**Affiliations:** 1 Michael Smith Laboratories and Department of Biochemistry and Molecular Biology, University of British Columbia, Vancouver, BC, Canada; 2 Division of Chemistry and Chemical Engineering, California Institute of Technology, Pasadena, CA, USA; 3 Department of Biochemistry, University of Illinois at Urbana–Champaign, Urbana, IL, USA; 4 Department of Chemical Engineering, University of Illinois at Chicago, Chicago, IL, USA; 5 Department of Chemistry and Biochemistry, University of California, Santa Barbara, CA, USA

## Abstract

Short-chain polyphosphate (polyP) is released from platelets upon platelet activation, but it is not clear if it contributes to thrombosis. PolyP has increased propensity to clot blood with increased polymer length and when localized onto particles, but it is unknown whether spatial localization of short-chain polyP can accelerate clotting of flowing blood. Here, numerical simulations predicted the effect of localization of polyP on clotting under flow, and this was tested *in vitro* using microfluidics. Synthetic polyP was more effective at triggering clotting of flowing blood plasma when localized on a surface than when solubilized in solution or when localized as nanoparticles, accelerating clotting at 10–200 fold lower concentrations, particularly at low to sub-physiological shear rates typical of where thrombosis occurs in large veins or valves. Thus, sub-micromolar concentrations of short-chain polyP can accelerate clotting of flowing blood plasma under flow at low to sub-physiological shear rates. However, a physiological mechanism for the localization of polyP to platelet or vascular surfaces remains unknown.

PolyP is an activator of blood coagulation through its ability to accelerate the activation of coagulation factors XII, XI, and V^1^,^2^, and by abrogating tissue factor pathway inhibitor (TFPI) function^3^,^4^. Long-chain polyP (hundreds to thousands of residues long) appears to be a much more potent activator of clotting, via activation of factor XII and the contact pathway, than short-chain polyp^5^,^6^,^7^. Short-chain polyP (60–100 phosphate residues long) is found in dense granules of human platelets and granules of mast cells (acidocalcisomes) and released upon their activation, while long-chain polyP occurs in microbes and some mammalian cells, such as in prostate cancer^7^,^8^,^9^,^10^. A characteristic of long-chain polyP is its ability to aggregate into particles, and this spatial localization may possibly contribute to its propensity to accelerate clotting^11^. It is less clear if there is a pathophysiological role for polyP released from human cells in thrombosis^3^. Short-chain endogenous polyP facilitates activation of FXII *in vitro*, albeit at supraphysiological concentrations^12^. It can also contribute to clotting *in vitro* under flow when tissue factor (TF) is present^13^. It is well-known that the local concentration of activators can profoundly influence their ability to initiate the clotting of blood^14^. Localization of polyP onto particles also accelerates coagulation under stagnant conditions^15^. Thus, we hypothesized that short-chain polyP may be a more effective activator when spatially localized onto surfaces, capable of accelerating clotting of flowing blood *in vitro* without participation of TF.

Initiation of blood coagulation is triggered when the local concentration of activators reaches a critical threshold, upon which the proteolytic cascade amplifies the local concentration of active enzymes to form a cross-linked fibrin mesh^16^,^17^. The spatial localization of activators to surfaces effectively increases their local concentration, allowing coagulation to be triggered with less total amount of activator^18^,^19^. Several activators have displayed this effect of spatial localization in microfluidic models of clotting, including TF, glass, and bacteria that activate prothrombin and factor X^20^. Flow influences coagulation in a variety of ways and enhances the effects of spatial localization^21^. Flow continuously strips clotting factors from catalytic surfaces, preventing activators from achieving a critical threshold and ultimately preventing clot initiation^22^,^23^. To accelerate clotting of flowing blood, greater amounts of activator need to be localized in order to achieve a higher local concentration^24^. In this study, we used numerical simulations and a microfluidic model of thrombosis to investigate whether the ability of localization to enhance clotting extends to short-chain polyP *in vitro* under flow. The shear rates used in this study range from low to sub-physiological (i.e. pathological) shear. These low shear rates mimic those typical of where thrombosis occurs in large veins and valves, such as in deep vein thrombosis (DVT) or those associated with airplane economy class syndrome^25^,^26^,^27^,^28^,^29^,^30^. This microfluidic model of thrombosis enabled clotting of plasma, or lack thereof, to be monitored over many hours in the absence of TF. In contrast to coagulation that occurs from acute injury to vessels, such as from puncture that exposes large amounts of TF, thrombosis may initiate over longer periods of time and can be potentiated by factor XII^2^,^31^,^32^,^33^,^34^,^35^. Our experiments were designed to determine if localization of physiologically-relevant concentrations of platelet-length polyP could contribute to coagulation *in vitro* at low to sub-physiological shear, but they do not validate whether or not localization of platelet-length polyP contributes to thrombosis *in vivo*.

## Results

### Numerical simulations predict the localization of polyP will increase its coagulability at low shear rates

**Figure 1:**
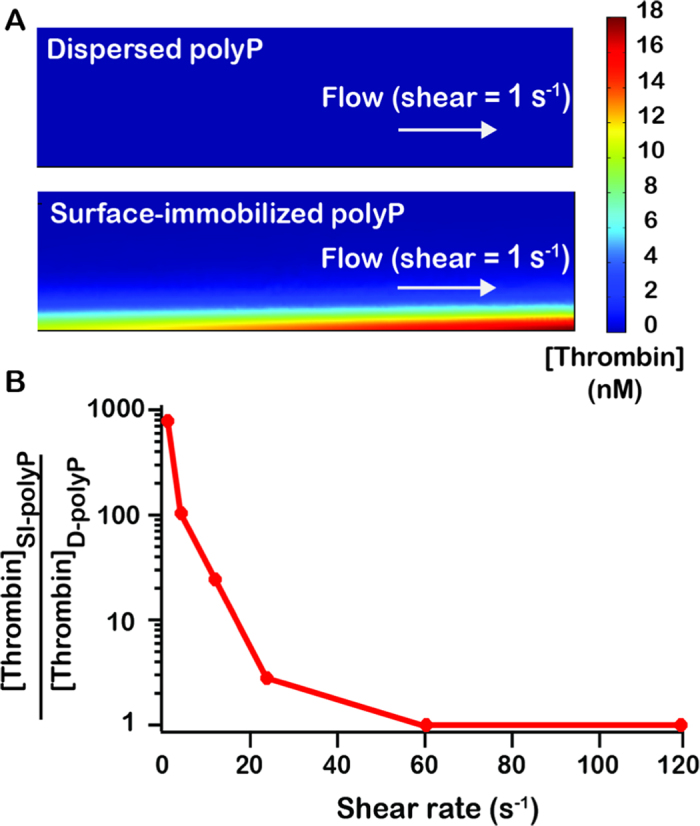
Numerical simulations predict localization of polyP accelerates thrombin production at low shear rates. Two-dimensional numerical simulations of the human blood coagulation cascade, comparing the generation of thrombin by polyP dispersed throughout a cylindrical channel versus polyP immobilized on the channel surface. The channel was 20 mm long with a radius of 2 mm. The overall number of polyP molecules was the same in all simulations (7.54 × 10^−9^ moles). (**A**) Plots show [thrombin], which is the sum of concentrations of thrombin and meizothrombin, for a two-dimensional longitudinal cut of the cylinder at 500 s into the simulation. (**B**) The fold difference in the maximum [thrombin] generated in the channel when polyP was surface-immobilized (SI-polyP) versus dispersed (D-polyP) at varying shear rates.

To initially examine how localizing polyP onto surfaces affects thrombin generation, we used a two-dimensional numerical simulation that considered diffusion, convection, and the rates of 41 reactions of the coagulation cascade (Supplementary Tables S1–3). An established kinetic model for the coagulation cascade was used with the addition of polyP_>1000_ in three reactions that were previously characterized in kinetic assays^1^,^4^,^5^,^7^,^20^. PolyP was either spatially localized onto the surface of a cylindrical channel or dispersed throughout its volume. Shear rates were from 1 s^−1^ to 120 s^−1^, a range that encompasses sub-physiological shear rates (<~10 s^−1^) and shear rates in the inferior vena cava, venous valves, and large veins^25^,^26^,^27^,^36^. When polyP was localized onto the surface of the channel with a shear rate of 1 s^−1^, the local thrombin burst was 782-fold higher than when an equal amount of polyP was dispersed throughout the volume (1.83 × 10^−8^ M versus 2.34 × 10^−11^ M) (Fig. 1A). The amount of polyP in the simulations was 7.54 × 10^−9^ mol, which equates to 30 μM (with respect to phosphate monomer) when the total volume of the simulation was considered. The resulting thrombin burst was a consequence of the higher local concentration of polyP, which led to increased positive feedback from the coagulation cascade. Simulations showed that differences in thrombin generation persisted over various shear rates, up to 60 s^−1^ (Fig. 1B). However, at a set distance, the difference decreased as shear rate increased, because thrombin was rapidly transported down-stream.

### Surface-immobilized polyP accelerates clotting of flowing blood plasma

**Figure 2:**
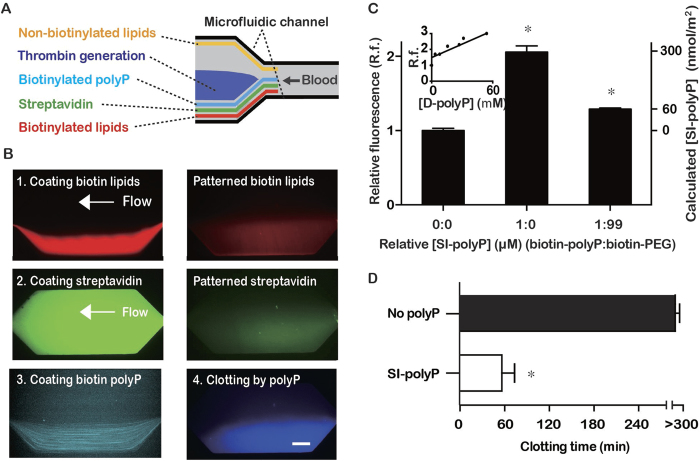
PolyP induces clotting of flowing blood plasma when localized on a surface at sub-physiological shear. (**A**) Schematic of biotinylated synthetic polyP (cyan) patterned onto the surface of half of a microfluidic channel, which induces production of thrombin and clotting (blue) of flowing blood plasma (grey). (**B**) Images of fluorescent-labeled agents flowing and patterned along one side of a microfluidic channel. Biotinylated lipids (tagged red) self-assembled on the channel wall. Non-biotinylated lipids (not tagged in these images) were simultaneously flowed and patterned on the other side of the chamber using laminar flow patterning. Then, streptavidin (tagged green) was flowed through and bound to the biotinylated lipids, followed by flowing biotinylated polyP labeled with DAPI (cyan), which bound streptavidin. A substrate (blue) for thrombin was activated, indicating initiation of clotting, selectively on patterned polyP_400_ (300 nmol/m^2^). Scale bar is 250 μm. (**C**) Quantifying of the amount of SI-polyP by measuring the fluorescence of DAPI bound to it. Channels with SI-polyP were compared to channels without polyP and to channels treated with polyP diluted with biotinylated PEG. Inset is a standard curve of known concentrations of solubilized D-polyP, which was used to calculate the surface concentration of SI-polyP in coated channels. (**D**) The clotting times of normal human plasma flowing through channels coated with polyP_400_ at a shear rate of 1 s^−1^. *p = < 0.01 compared to controls without polyP. Data indicate mean ± SEM, *n* = 3.

To determine if SI-polyP was able to accelerate clotting of flowing blood plasma, synthetic polyP_400_ was immobilized onto the walls of microfluidic channels (Fig. 2A). Half of each chamber was patterned with biotinylated lipids followed by an excess of streptavidin (Fig. 2B). Biotinylated-polyP_400_ was then flowed through the channel, becoming immobilized onto streptavidin. The surface concentration of SI-polyP_400_ was varied by diluting biotinylated-polyP_400_ in a solution of biotin-PEG before coating the channel. The concentration of polyP was determined by DAPI staining. Fluorescence intensities from known concentrations of stained D-polyP, which was soluble and dispersed throughout the channel, were used to generate a standard curve and used to calculate the surface concentration of SI-polyP (Fig. 2C). The surface concentration of SI-polyP was 300 nmol/m^2^, and could be decreased to 60 nmol/m^2^ by diluting with biotin-PEG. To test the ability of patterned polyP to induce clotting, platelet-poor human plasma was flowed through the chambers. Based on the simulation data, we tested the lowest shear (1 s^−1^) as it was predicted to have the largest effect on thrombin generation and therefor clotting. A range of shear rates are explored in later experiments. The plasma clotted selectively on areas with immobilized polyP_400_ (300 nmol/m^2^) in 50–70 min at a shear rate of 1 s^−1^. No clotting was observed over 5 hr in channels without polyP_400_ (Fig. 2D). All polyP concentrations are reported in terms of phosphate monomer.

### Measuring clot times simultaneously at various shear rates

**Figure 3:**
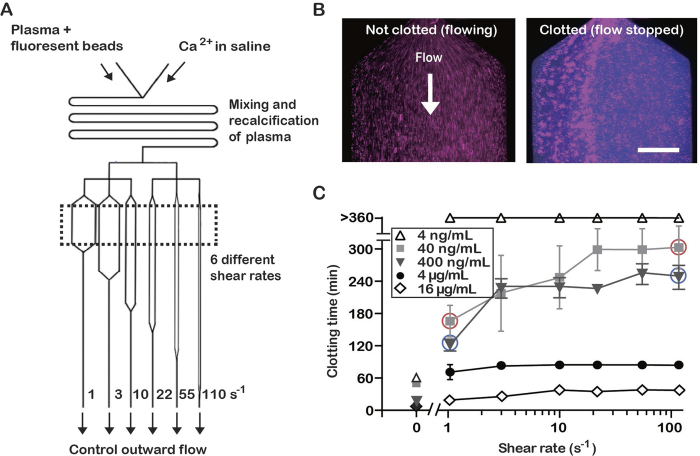
The microfluidic system used to measure clotting over a range of shear rates. (**A**) Schematic of the microfluidic system. Box (dashed lines) indicates the region where shear rates were varied and clot times were measured. (**B**) Fluorescence images showing that clotting was detected by the cessation of flow of tracer beads (pink) and by the cleavage of a substrate for thrombin (blue). Scale bar is 250 μm. (**C**) Assessing the range of clotting times in this flow system by adding various concentrations of FVIIa to the plasma. Data points indicate mean ± SEM, *n* = 3–4. Red circles indicate *p* = <0.05 between the data points, and blue circles indicate *p* = <0.01 between the data points.

A microfluidic system containing six regions with varying shear rates was used to measure clot times of flowing blood plasma (Fig. 3A). The range of shear rates was 1–110 s^−1^, which encompasses physiological shear rates which occur in the inferior vena cava, venous valves, and large veins; as well as, sub-physiological shear rates (<~10 s^−1^) that occur in pathological contexts^25^,^26^,^27^,^28^,^36^. These calculated shear rates were within 3–8% of the values obtained by measuring the flow velocity of micro particles by florescence microscopy. Clotting was monitored by visualizing the movement of fluorescent tracer beads specifically in the shear chambers, which became immobilized in clotted regions, and by a fluorogenic peptide substrate, which fluoresced when cleaved by thrombin during clotting (Fig. 3B). To characterize and determine the range of clot times of flowing blood plasma in the microfluidic system, coagulation factor VIIa (FVIIa) was used, and added to plasma at a range of concentrations (Fig. 3C). FVIIa does not circulate in plasma in appreciable amounts physiologically (~1% of total FVII circulates as FVIIa)^37^, but is administered during severe hemorrhage in some cases to aid in hemostasis at doses of 90 to 270 μg/kg, which roughly corresponds to 1 to 4 μg/mL in plasma^38^,^39^. In the device, plasma containing 16 μg/mL of FVIIa clotted in approximately 20–40 min, plasma containing 4 μg/mL of FVIIa clotted in approximately 60 min, and plasma containing 4 ng/mL did not clot within 6 hr. Intermediate clotting times occurred with concentrations of 400 ng/mL and 40 ng/mL and were dependent on shear rate. Clot formation always occurred from the channel wall, crudely mimicking how physiological thrombus formation occurs from the walls of blood vessels and is shear-dependent^40^.

### Short-chain polyP accelerates clot formation faster when surface-localized than when dispersed in nanoparticles or in solution

**Figure 4:**
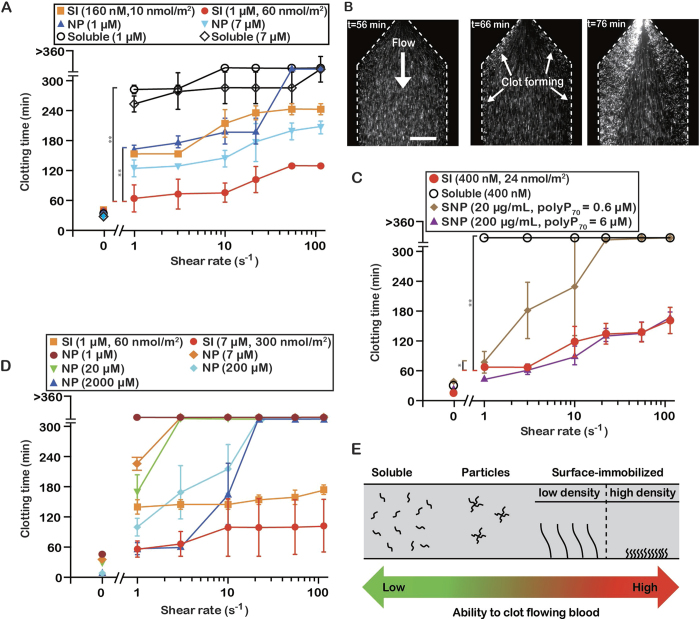
PolyP accelerates clotting best when spatially localized onto surfaces, compared to soluble polyP and nanoparticles of polyP. (**A**) Clotting times of plasma by polyP_160_ at varying shear rates, comparing three states of polyP_160_: solubilized, self-assembled nanoparticles, and surface-immobilized. (**B**) Time-lapse images showing SI-polyP_160_ initiating clotting (detected by non-flowing beads) from the channel wall (dashed lines). Scale bar is 250 μm. (**C**) Comparing three states of polyP_70_: solubilized, surface-immobilized onto the microfluidic channels, and immobilized onto silica nanoparticles. Clotting tendencies of plasma containing silica nanoparticles coated with polyP_70_ (SNP-polyP_70_) compared to soluble and surface-immobilized polyP_70_ under shear in the microfluidic device. (**D**) Comparing two states of long-chain polyP: surface immobilized polyP_400_ and nanoparticles of self-assembled polyP_>1000_. (**E**) Schematic summarizing the relationship between spatial distribution of polyP and the acceleration of clotting in the above experiments. Data points indicate mean ± SEM, *p < 0.001, **p < 0.0001, *n* = 3–4. Statistical analysis represents comparisons between whole curves.

PolyP_160_ was previously demonstrated to be a weak initiator of the contact pathway, but we examined the hypothesis that spatially localizing polyP_160_ onto a surface (SI-polyP_160_) would enhance its ability to contribute to clot formation compared to polyP_160_ dispersed as nanoparticles (NP-polyP_160_) or in solution (D-polyP_160_, Fig. 4A). With NP-polyP_160_ (1 μM, 250 nm diameter, Supplementary Fig. S1), clotting occurred in approximately 170 min and 200 min at a shear rate of 1 s^−1^ and 22 s^−1^ respectively. When a similar amount of polyP_160_ was localized onto the channel surface, clotting occurred significantly faster than both NP-polyP_160_ and D-polyP_160_. Clotting initiated from the parallel channel shear chamber walls, or in areas where the channel expanded from high to very low shear, and progressively grew outwards (Fig. 4B). Clotting with D-polyP_160_ (1 μM) was 4- to 2.8-fold slower than SI-polyP_160_ and 1.6- to 0-fold slower than NP-polyP_160_ at all shear rates. Overall, clotting occurred fastest with SI-polyP_160_ than dispersed polyP_160_ in either soluble or NP forms, even with 6–43 fold less SI-polyP_160_ in the channels.

### Platelet-length polyP can accelerate clotting when surface-localized

The concentration of polyP is approximately 1.1 mM in platelets, where it is stored in platelet dense granules, and can reach up to 2–7 μM in blood upon platelet activation^41^,^42^. To test whether synthetic polyP similar in length to those found in human platelets can clot flowing blood at physiological concentrations, polyP_70_ was tested (Fig. 4C). Soluble polyP_70_ (D-polyP_70_) at 400 nM did not accelerate clotting of flowing blood plasma at the shear rates tested. In contrast, an equivalent amount of SI-polyP_70_ substantially accelerated clotting, to 70 min and 160 min at shear rates of 1 s^−1^ and 110 s^−1^ respectively. The amount of SI-polyP_70_ used corresponded to a surface concentration of 24 nmol/m^2^ and a total concentration of around 400 nM in the volume of the channel. Initiation time of clotting by SI-polyP_70_ was dependent on FXII (Supplementary Fig. S2).

SI-polyP_70_ and D-polyP_70_ could not be directly compared to self-assembled nanoparticles of polyP_70_ (NP-polyP_70_), because the solubility of polyP_70_ is greater than longer chain polyP, and NP-polyP_70_ was not stable. Alternatively, we tested a second formulation of polyP nanoparticles, where polyP_70_ was coated on silica nanoparticles (SNP-polyP_70_)^43^. When SNP-polyP_70_ was added to plasma at varying shear, clotting occurred in approximately 70 min to 160 min at 200 μg/mL and 80 min to >360 min at 20 μg/mL. These masses of SNP-polyP_70_ corresponded to concentrations of polyP_70_ of 6 μM and 0.6 μM, respectively, but include both polyP_70_ and silica. Silica is also an activator of factor XII, so an equal comparison between SI-polyP_70_ and SNP-polyP_70_ cannot be made. Nevertheless, the clotting times of SI-polyP_70_ (400 nM) were significantly faster than 20 μg/mL of a SNP-polyP_70_, and were nearly identical to 200 μg/mL of a SNP-polyP_70_ even though there was a 15-fold lower concentration of polyP_70_.

### Clotting by long-chain polyP is also enhanced by surface localization

To understand if the effect of surface localization extends to long-chain polyP, we tested a range of concentrations of long-chain polyP either surface localized (SI-polyP_400_) or dispersed as nanoparticles (NP-polyP_>1000_). PolyP_>1000_ naturally self-assembles, localizing into nanoparticles of 150 ± 30 nm in diameter in solutions containing Ca^2+^ at low millimolar concentrations^11^. It is a known activator of clotting under static conditions when dispersed throughout plasma^3^. We compared NP-polyP_>1000_ to SI-polyP_400_, rather than SI-polyP_>1000_, because surface patterning of polyP requires biotinylation of the polyP chains, and the biotinylation procedure caused degradation of long chain-lengths of polyP. When plasma was flowed over SI-polyP_400_, clotting occurred in approximately 60 min to 100 min at 7 μM and 140 min to 170 min at 1 μM at the shear rates examined (Fig. 4D). The clot times using NP-polyP_>1000_ demonstrated robust shear- and concentration-dependence at 2000, 200, 20, 7 and 1 μM. NP-PolyP_>1000_ was most potent at 2000 μM, initiating clotting at 60 min at 1 and 3 s^−1^, although requiring 285-fold more phosphate to match the propensity of SI-polyP_400_ to clot flowing plasma under the same conditions.

## Discussion

Together, these data show that the spatial localization of synthetic polyP onto surfaces affects its ability to activate clotting under flow (Fig. 4E). Short-chain polyP polymers (polyP_160_ and polyP_70_) greatly accelerated clotting of flowing blood plasma at low to sub-physiological shear when surface-localized onto the walls of microfluidic chambers compared to when they are dispersed (nanoparticle or soluble forms). Soluble short-chain polyP only clotted stagnant blood (near-zero flow) in our experiments, and clotting did not occur within a span of hours even at sub-physiological shear rates. Localization of polyP onto the surface of channels showed the greatest activity overall. The concentration at which SI-polyP_70_ accelerated clotting *in vitro* is well-within the range of amounts of polyP released into plasma following platelet activation. Although it is not known if polyP localizes to cell surfaces or thrombi, or to the extent polyP contributes to physiological or pathophysiological coagulation, it is important to identify scenarios in which polyP could potentially elicit a role. These results propose that if polyP can surface-localize it may contribute to clotting at sub-physiological shear following platelet activation, but further *in vitro* and *in vivo* experiments are necessary to verify that this is a potential mechanism.

Remarkably, comparing SI-polyP_70_, SI-polyP_160_, and SI-polyP_400_, to each other shows that short-chain polyP could match the propensity of longer chain polyP to accelerate clotting under flow. SI-polyP_70_ accelerated clotting to a similar extent as SI-polyP_160_ and SI-polyP_400_ with a lower concentration of phosphate. Although clotting times were similar between them with respect to surface coverage of full-length polymers. This is likely because shorter chains have high surface coverage relative to the amount of monomer. Thus, clotting occurred faster with both increasing surface concentration of phosphate and increasing surface coverage.

The simulations predicted the trend observed *in vitro*. Localization creates high local concentrations of polyP, and in the numerical simulations this led to larger thrombin bursts due to increased positive feedback from the coagulation cascade. The simulations included polyP binding and inhibiting TFPI and accelerating activation of factors V and XI, which all occur in plasma. The mechanism is likely contact system mediated as under stagnant conditions FXII contributed to initiation of clotting by polyP, but we did not test this further in flow experiments. It was recently shown that short-chain polyP could complex with FXII *in vitro* to allostericly induce its activation at high polyP concentrations of 70–130 μM^12^. This polyP-induced activation of FXII was enhanced in the presence of zinc ions, which is known to bind robustly to both FXII and PolyP^3^,^12^. Short-chain polyP can also contribute to clotting independently of FXII when TF is present^13^. The results here, without TF, indicate that localization can further increase the propensity of short-chain polyP to clot blood plasma.

In these microfluidic experiments, shear rate and concentrations of either FVIIa or polyP influenced the clotting times over several hours. The shear rates mimicked the shear rates that are typical in large veins and valves; as well as, pathological shear which occurs it the context of thrombosis. The reported clotting times appear very long compared to clotting times in most *in vitro*, stagnant clotting assays, which occur in seconds to minutes. However, residence time of plasma in the microfluidic chambers was only ~10 sec, with plasma being continuously transported into and out of the chamber, and thus the rate of clotting cannot be directly compared to stagnant clotting assays. Long clot times were possible in this device, compared to most other flow systems, because platelet-poor plasma was recalcified on the device and because TF was not included^44^. The observed clotting times were much slower than what is typical in acute hemostasis and at high concentrations of TF, but they were within the time-frame that formation and growth of thrombi occurs inside veins and regions of low shear^31^,^32^,^33^. Thrombosis, in contrast to hemostasis, can involve progressive and gradual clot growth, where there is much less TF but increased contribution of factors XI and XII^2^,^45^. The clotting times measured in this microfluidic system are more representative of clotting times that would occur during thrombosis inside intact veins, rather than punctured vessels or stagnant clotting assays. In addition, the shear rates used in our microfluidic model include the rates which occur in large veins. Though platelets appear to contribute more to arterial thrombi than venous thrombi, they also contribute to venous thrombosis^46^,^47^. For example, antiplatelet drugs have also been beneficial in treating venous thrombosis^47^,^48^,^49^.

For several concentrations and chain-lengths of polyP, it was not possible to make equivalent comparisons between SI-polyP, NP-polyP, SNP-polyP, and D-polyP, because the chain-length and concentration are important determinants of whether polyP self-assembles into particles or remains soluble. The solubility of short-chain polyP is greater than long-chain polyP, but solubility also depends on the concentration of polyP and Ca^2+^. For example, polyP_160_ can be formulated to be soluble or to form NP-polyP by varying the concentration of polyP and Ca^2+ ^11. We used concentrations of polyP_160_ well below its limit of solubility. PolyP_160_ was first dissolved into water and then diluted into plasma. When added to citrated plasma, soluble polyP likely remained dissolved, as plasma has insufficient free divalent cations to facilitate nanoparticle formation^11^. Once plasma is recalcified, polyP likely remains protein-bound even in the presence of low millimolar amounts of ionic calcium, at least for the 35 sec that it is present in the microfluidic devices (Supplementary Fig. S3)^11^,^50^. In contrast, NP-polyP were formed by precipitating polyP_160_ in 5 mM Ca^2+^, generating nanoparticles that were stable for over 6 hr, as measured by dynamic light scattering. NP-polyP was diluted in the calcium-saline solution that mixed with blood plasma inside the microfluidic devices to keep the nanoparticles intact. The stability of NP-polyP in plasma is unknown; however, NP-polyP was initially prepared under supersaturated conditions, and the solubility of NP-polyP displays hysteretic behavior^11^. Thus, a large portion of NP-polyP, once formed, likely remained as NPs in the microfluidic devices. Although synthetic polyP was used in these experiments, natural polyP is also typically bound to calcium^51^.

In summary, this work shows that spatial localization of synthetic polyP, including short-chain polyP, increases its propensity to accelerate clotting of blood plasma at low to sub-physiological shear. The observed clotting times were much slower than what is typical in hemostasis, but they were within the time-frame that thrombosis occurs inside veins, particularly post-operative deep vein thrombi, which form over a period of days^32^,^33^,^52^. The experiments were designed solely to test if surface-localization of short-chain polyP accelerates clot formation under flow, at venous and sub-physiological shear rates. An important observation from this was that when localized, short-chain polyP could match the ability of long-chain polyP to accelerate clotting. The concentration required to accelerate clotting is markedly reduced when polyP is spatially localized onto surfaces, and to a lesser extent, into particles, even under flow and without TF. These biophysical insights provide a potential biophysical mechanism by which platelet-length polyP could contribute to thrombosis in regions of low shear, but further work is required to validate if this mechanism could indeed extend to *in vivo* scenarios. This effect of localization may potentially contribute to clotting at higher shear when TF is present^13^. Although these *in vitro* results, in an artificial flow system, support the notion that platelet-derived polyP could contribute to coagulation *in vivo*, the flow system used here does not include many factors that normally regulate clotting, such as platelets, platelet-derived polyP, red blood cells, immune cells, endothelium and other soluble factors. For these reasons, appropriate *in vivo* models are necessary to verify whether platelet-derived polyP and its spatial localization contributes to clot formation and thrombosis.

## Materials and Methods

### Numerical Simulations

Thrombin generation was modeled with the *Transport of Diluted Species* module of Comsol Multiphysics 4.4 by adding diffusion and convection to a previously reported kinetic model^20^. Changes to the model included the addition of three rate equations to describe the activity of polyP: 1) the binding and inhibition of TFPI; TFPI + polyP ↔ TFPI-polyP; k_on_ = 4.0 × 10^5^ M^−1^s^−1^, k_off_ = 1.0 × 10^−2^ s^−1^, 2) the activation of factor V; V + Xa + polyP → Va + Xa + polyP; k = 8.0 × 10^12^ M^−2^s^−1^, 3) the activation of factor XI; XI + IIa + polyP → XIa + IIa + polyP; k = 8.8 × 10^9^ M^−2^s^−1 ^1,4,5,7,20. The diffusion coefficient for all soluble species was 5 × 10^−11^ m^2^/s and the velocity profile varied with the shear rate, 
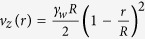
, where *v*_*z*_ is the velocity in the axial direction at each radial coordinate *r, R* is the cylinder radius, and *γ*_*w*_ is the shear rate at the cylinder wall. The chemical species were flowed into a cylindrical geometry of radius 2 mm and length 20 mm. For each shear rate, [thrombin] was sampled after the incoming flow had displaced the channel volume 12.5 times. Both the experiments and simulations were performed in the same mass-transfer regime (*Pe* ≫ 1 and *Gz* > 3000).

### Preparing soluble polyP (D-polyP), self-assembled polyP nanoparticles (NP-polyP), polyP-coated silica nanoparticles (SNP-polyP), and surface-immobilized polyP (SI-polyP)

To make D-polyP, polyP was solubilized and diluted first in water and then added to citrated plasma (frozen citrated normal control plasma, Affinity Biologicals Inc.) prior to entering the microfluidic device. NP-polyP was generated as described previously^11^. Briefly, soluble polyP was added to a calcium solution (10 mM polyP, 5 mM CaCl_2_, 8 mM Tris, pH 6.0) followed by vortexing, during which polyP self-assembled into nanoparticles tightly bound to Ca^2+^ cations^11^. The formation and size of the nanoparticles were verified after adding them to this calcium solution by observing the scattering intensity and hydrodynamic diameter, as measured by dynamic light scattering (Zetasizer Nano ZSP, Malvern Instruments). NP-polyP formulations were added and diluted in calcium-saline solution, rather than the citrated-plasma, prior to entering the microfluidic device. The NP-polyP were stable for over 6 hr in these solutions. SNP-polyP_70_ were made by covalently attaching polyP_70_ onto silica nanoparticles as previously described^43^. Synthetic PolyP was generated by solubilization from Maddrell salts and biotinylated as previously described^7^,^50^. Synthetic polyP has been previously characterized, including its chain length, counterions and clotting activity^7^,^11^. Long-chain NP-polyP contained a heterogeneous preparation of very long, non-biotinylated polyP polymers ranging from around 200 mers to 1300 mers, referred to here as NP-PolyP_>1000_. Some experiments with SI-polyP employed heterogeneous long-chain biotinylated polyP consisting of chains 50 to 400 units in length, referred to here as biotin-polyP_400_. Some experiments employed fractionated material of narrower sizes (polyP_70_ and polyP_160_)^7^. All polyP concentrations are stated with respect to the concentration of phosphate monomer.

### Preparing microfluidic devices with SI-polyP

Microfluidic devices were prepared from polydimethylsiloxane (PDMS) as previously described^53^. Channel dimensions are listed as follows (length × width, 125 μm height for all channels): 1.67 mm × 1000 μm (1 s^−1^), 3.33 mm × 500 μm (3 s^−1^), 5.83 mm × 286 μm (10 s^−1^), 8.33 mm × 200 μm (22 s^−1^), 12.50 mm × 133 μm (55 s^−1^), 16.67 mm, 100 μm (110 s^−1^). The devices were incubated in saline and kept under vacuum overnight to hydrate and remove air from the channels. Devices remained soaked in saline throughout the experiment to aid in coating the surfaces with lipids, and to reduce convective flow during experiments in the absence of flow. The devices were coated with phosphatidylcholine (PC) vesicles to prevent activation of clotting on the PDMS surface. In devices that were not coated with SI-polyP, vesicles were prepared with egg PC (Avanti Polar Lipids, Alabaster, USA) and fluorescent Texas Red 1,2-Dihexadecanoyl-*sn*-Glycero-3-Phosphoethanolamine (DHPE) (Invitrogen) in a 99.5:0.5 molar ratio. Lipids were extruded through a 100 nm membrane using a Lipex Thermobarrel Extruder (Northern Lipids, Burnaby, Canada). The vesicle solution (10 mg/mL in dH_2_O) were flowed through microfluidic channels at a rate of 1 μl/min for 15 min and rinsed out with saline. The coating of PC on the channels was stable for at least 10 hours (Supplementary Fig. S4). For devices where polyP was surface-immobilized, the channel was first coated with biotinylated lipids (1 μl/min, 15 min) and rinsed out with saline. To prepare biotinylated vesicles, 1-oleoyl-2-[12[biotinyl(aminododecanoyl)]-sn-glycero-3-phosphocholine (biotinylated-PC, Avanti Polar Lipids) was mixed with Egg PC and Texas Red DHPE in a molar ratio of 5.0:94.5:0.5 and extruded. Next, streptavidin (100 μg/mL) conjugated to Alexa Fluor^®^ 488 (Molecular Probes, Inc.) was flowed through the device (1 μl/min, 40 min) and then rinsed with saline to wash away the unbound, excess streptavidin. Finally, a solution of biotin-polyP (50 μg/mL) and biotinylated-polyethylene glycol (biotin-PEG) (either 0 or 99 molar equivalents to biotin-polyP) was flowed through the device (1 μl/min, 40 min), binding to the patterned streptavidin followed by a saline rinse, which resulted in SI-polyP being selectively patterned on the walls of the microfluidic device shear chambers. Liposomes and saline were flowed into the device through a combination of inlet and outlet channels to achieve laminar flow patterning, such that the parallel streams of fluids were at low Reynolds number (≪1) and maintained sharp boundaries and excluded the possibility of turbulent flow^54^,^55^. This patterning allowed specific channel walls of the device to be coated, either all channels in the device, the channels in the shear chambers, or one wall of the chambers. To measure the amount of polyP, it was stained by flowing DAPI (40 μg/mL in 15 mM Tris acetate, 300 mM NaCl, 30 mM EDTA, and 0.02% NaN_3_) into the device. Thrombin generation during clotting was detected by adding 125 μg/mL of fluorescent peptide substrate for thrombin (Boc-Val-Pro-Arg-4-methylcoumaryl-7-amide, Peptide Institute Inc.) into normal plasma.

### Flowing plasma and calcium into devices and measuring clotting

Flow rates were controlled using a syringe pump (Harvard Apparatus PHD 2000) by withdrawing solutions out of the outlets of the device at a rate of 1 μl/min. Shear rates in different channels were controlled by the width of each channel, while the residence time of plasma within the shear chambers were kept constant (~10 sec) by varying their respective lengths. Tubing connected to the outlets of the device were charged with 50 μl of Egg PC vesicles to prevent clotting from initiating in the tubing or syringes. A solution of sodium citrate (10 mM in dH_2_O) was initially pulled into both inlet channels to wash out the device and further charge the outlet tubing. Normal citrated human plasma (7 mM citrate) and calcium-saline solution (40 mM CaCl_2_ and 90 mM NaCl) were simultaneously pulled into the device and mixed at a ratio of 3:1 to recalcify the plasma, yielding a final free calcium concentration of 4–5 mM^56^. To measure clotting times, fluorescent beads (2.5 μg/mL, Fluoresbrite Plain YG 1.0 Micron Microsphere, Polysciences Inc.), and in some experiments 125 μg/mL fluorescent thrombin substrate, were mixed into the plasma and time-course imaging of each channel was performed using an epifluorescence microscope (Leica DMI6000B). The fluorescent beads did not influence clotting times (Supplementary Fig. S5). Clotting was determined by the immobilization of the fluorescent beads and in some experiments also by the generation of blue fluorescence upon cleavage of the thrombin substrate. In experiments where the effect of nanoparticle polyP (NP-polyP) on clotting was tested, the activators were mixed with the calcium solution prior to entering the device. For experiments with soluble polyP (D-polyP), polyP was added to the plasma instead to prevent nanoparticle formation. For experiments at zero shear, normal or congenital FXII-deficient plasma (Geroge King Bio-Medical, Inc.) and calcium were mixed together immediately before flowing them into the device and blocking all outlets to create stagnant plasma.

## Additional Information

**How to cite this article**: Yeon, J. H. *et al*. Localization of Short-Chain Polyphosphate Enhances its Ability to Clot Flowing Blood Plasma. *Sci. Rep.* **7**, 42119; doi: 10.1038/srep42119 (2017).

**Publisher's note:** Springer Nature remains neutral with regard to jurisdictional claims in published maps and institutional affiliations.

## Supplementary Material

Supplementary Information
