# Supplementary material for: Localization of Short-Chain Polyphosphate Enhances its Ability to Clot Flowing Blood Plasma
Source: Sci Rep. 2017 Feb 10;7:42119. doi: 10.1038/srep42119 (PMC5301195; doi:10.1038/srep42119)
Supplement: Supplementary Information [file srep42119-s1.pdf]

LOCALIZATION OF SHORT-CHAIN POLYPHOSPHATE ENHANCES ITS ABILITY TO CLOT FLOWING BLOOD PLASMA

Ju Hun Yeon[1¶], Nima Mazinani[1¶], Travis S. Schlappi[2], Karen Y. T. Chan[1], James R. Baylis[1], Stephanie A. Smith[3], Alexander J. Donovan[4], Damien Kudela[5], Galen D. Stucky[5], Ying Liu[4], James H. Morrissey[3], Christian J. Kastrup[1*]

1 Michael Smith Laboratories and Department of Biochemistry and Molecular Biology, University of British Columbia, Vancouver, BC, Canada, **2** Division of Chemistry and Chemical Engineering, California Institute of Technology, Pasadena, CA, USA, **3** Department of Biochemistry, University of Illinois at Urbana–Champaign, Urbana, IL, USA, **4** Department of Chemical Engineering, University of Illinois at Chicago, Chicago, IL, USA, **5** Department of Chemistry and Biochemistry, University of California, Santa Barbara, CA, USA

¶ These authors contributed equally to this work.

* ckastrup@msl.ubc.ca

Supplementary Table S1: Reactions and rate constants used in the simulations for Fig. 1.

Rxn	Model Expressions	k_1	k_{-1}	k_{cat}	References
1	Xa + VII → Xa + VIIa	$1.3 \cdot 10^7$ $M^{-1}s^{-1}$			(20)
2	IIa + VII → IIa + VIIa	$2.3 \cdot 10^4$ $M^{-1}s^{-1}$			(20)
3	II + Xa → IIa + Xa	$7.5 \cdot 10^3$ $M^{-1}s^{-1}$			(20)
4	IIa + VIII → IIa + VIIIa	$2.0 \cdot 10^7$ $M^{-1}s^{-1}$			(20)
5	VIIIa + IXa ↔ IXaVIIa	$1.0 \cdot 10^7$ $M^{-1}s^{-1}$	$5.0 \cdot 10^{-3}$ s^{-1}		(20)
6	IXaVIIa + X ↔ IXaVIIaX → IXaVIIa + Xa	$1.0 \cdot 10^8$ $M^{-1}s^{-1}$	$1.0 \cdot 10^{-3}$ s^{-1}	8.2 s^{-1}	(20)
7	VIIIa ↔ VIIIa1L + VIIIa2	$6.0 \cdot 10^{-3}$ s^{-1}	$2.2 \cdot 10^4$ $M^{-1}s^{-1}$		(20)
8	IXaVIIIaX → VIIIa1L + VIIIa2 + X + IXa	$1.0 \cdot 10^{-3}$ s^{-1}			(20)
9	IXaVIIIa → VIIIa1L + VIIIa2 + IXa	$1.0 \cdot 10^{-3}$ s^{-1}			(20)
10	IIa + V → IIa + Va	$2.0 \cdot 10^7$ $M^{-1}s^{-1}$			(20)
11	Xa+ Va ↔ XaVa	$7.5 \cdot 10^3$ $M^{-1}s^{-1}$	0.2 s^{-1}		(20)
12	XaVa + II ↔ XaVaII → XaVa + mIIa	$1.0 \cdot 10^8$ $M^{-1}s^{-1}$	103 s^{-1}	63.5 s^{-1}	(20)
13	XaVa + mIIa → XaVa + IIa	$1.5 \cdot 10^7$ $M^{-1}s^{-1}$			(20)
14	Xa + TFPI ↔ XaTFPI	$9.0 \cdot 10^5$ $M^{-1}s^{-1}$	$3.6 \cdot 10^{-4}$ s^{-1}		(20)
15	Xa + ATIII → XaATIII	$1.5 \cdot 10^{-3}$ $M^{-1}s^{-1}$			(20)
16	mIIa + ATIII → mIIaATIII	$7.1 \cdot 10^3$ $M^{-1}s^{-1}$			(20)
17	IXa + ATIII → IXaATIII	$4.9 \cdot 10^2$ $M^{-1}s^{-1}$			(20)
18	IIa + ATIII → IIaATIII	$7.1 \cdot 10^3$ $M^{-1}s^{-1}$			(20)
19	XIIa + XII ↔ XIIaXII → XIIa + XIIa	$1.0 \cdot 10^8$ $M^{-1}s^{-1}$	750 s^{-1}	$3.3 \cdot 10^{-2}$ s^{-1}	(20)
20	XIIa + PK ↔ XIIaPK → XIIa + K	$1.0 \cdot 10^8$ $M^{-1}s^{-1}$	$3.6 \cdot 10^3$ s^{-1}	40 s^{-1}	(20)
21	XII + K ↔ XIIK → XIIa + K	$1.0 \cdot 10^8$ $M^{-1}s^{-1}$	45.3 s^{-1}	5.7 s^{-1}	(20)
22	PK + K → K + K	$2.7 \cdot 10^4$ $M^{-1}s^{-1}$			(20)
23	K → KInhibited	$1.1 \cdot 10^{-2}$ s^{-1}			(20)
24	XIIa + C1inh → XIIaC1inh	$3.6 \cdot 10^3$ $M^{-1}s^{-1}$			(20)
25	XIIa + ATIII → XIIaATIII	21.6 $M^{-1}s^{-1}$			(20)

26	XI + IIa ↔ XIIIa → XIa + IIa	$1.0\cdot10^{8}$ M^{-1}s^{-1}	5.0 s^{-1}	$1.3\cdot10^{-4}$ s^{-1}	(20)
27	XIIa + XI ↔ XIIaXI → XIIa + XIa	$1.0\cdot10^{8}$ M^{-1}s^{-1}	200 s^{-1}	$5.7\cdot10^{-4}$ s^{-1}	(20)
28	XIa + XI → XIa + XIa	$3.19\cdot10^{6}$ M^{-1}s^{-1}			(20)
29	XIa + ATIII → XIaATIII	$3.2\cdot10^{2}$ M^{-1}s^{-1}			(20)
30	XIa + C1inh → XIaC1inh	$1.8\cdot10^{3}$ M^{-1}s^{-1}			(20)
31	XIa + a1AT → XIaa1AT	$1.0\cdot10^{2}$ M^{-1}s^{-1}			(20)
32	XIa + a2AP → XIaa2AP	$4.3\cdot10^{3}$ M^{-1}s^{-1}			(20)
33	XIa + IX ↔ XIaIX → XIa + IXa	$1.0\cdot10^{8}$ M^{-1}s^{-1}	41 s^{-1}	7.7 s^{-1}	(20)
34	IXa + X ↔ IXaX → IXa + Xa	$1.0\cdot10^{8}$ M^{-1}s^{-1}	0.64 s^{-1}	$7.0\cdot10^{-4}$ s^{-1}	(20)
35	Xa + VIII ↔ XaVIII → Xa + VIIIa	$1.0\cdot10^{8}$ M^{-1}s^{-1}	2.1 s^{-1}	0.023 s^{-1}	(20)
36	VIIa + IX ↔ VIIaIX → VIIa + IXa	$1.0\cdot10^{8}$ M^{-1}s^{-1}	0.9 s^{-1}	$3.6\cdot10^{-5}$ s^{-1}	(20)
37	VIIa + X ↔ VIIaX → VIIa + Xa	$1.0\cdot10^{8}$ M^{-1}s^{-1}	210 s^{-1}	$1.6\cdot10^{-6}$ s^{-1}	(20)
38*	polyP + TFPI ↔ polyPTFPI	$4.0\cdot10^{5}$ M^{-1}s^{-1}	$1.0\cdot10^{-2}$ s^{-1}		(4),(5)
39*	V + Xa + polyP → Va + Xa + polyP	$8.0\cdot10^{12}$ M^{-2}s^{-1}			(20),(4)
40*	XI + IIa + polyP → XIa + IIa + polyP	$8.8\cdot10^{9}$ M^{-2}s^{-1}			(5)

*Reactions 38-40 are bulk reactions for dispersed polyP simulations (D-polyP) and surface reactions for the surface-immobilized polyP simulations (SI-polyP).

Reaction 38: polyP abrogates TFPI function (4).

For polyP binding to TFPI, similar rate constants were used as when polyP binds and releases from other plasma proteins. The association rate constant for the polyP reaction with IIa, XI, and XIa ranges from $1.64\cdot10^{6}$ M^{-1}s^{-1} to $5.12\cdot10^{6}$ M^{-1}s^{-1} (5); thus, $1.0\cdot10^{6}$ M^{-1}s^{-1} was chosen for $k_{1,38}$. The disassociation rate constant for the polyP reaction with IIa, XI, and XIa ranges from $1.05\cdot10^{-2}$ s^{-1} to $7.71\cdot10^{-2}$ s^{-1} (5); thus, $1.0\cdot10^{-2}$ s^{-1} was chosen for $k_{-1,38}$.

Reaction 39: polyP accelerates V activation by Xa (4).

The activation of V to Va by IIa occurs with a rate constant ($k_{1,10}$) of $2.0 \cdot 10^{7}$ $M^{-1}s^{-1}$ (see Reaction 10 in Table S1) (20). The reaction is $V + IIa \rightarrow Va + IIa$ and the rate law is:

$$\frac{d[Va]}{dt} = k_{1,10}[V][IIa]$$

PolyP accelerates the activation of V by Xa; we assume this occurs with a similar rate as that of V activation by IIa. The reaction would be $V + Xa + polyP \rightarrow Va + Xa + polyP$ and the rate law would be:

$$\frac{d[Va]}{dt} = k_{1,39}[V][Xa][polyP]$$

To approximate the rate constant, we assume that $k_{1,10} \sim k_{1,39}[polyP]$. With a typical $[polyP]{\sim}1\mu M$, this means that $k_{1,39} \sim 2.0 \cdot 10^{13} M^{-2}s^{-1}$.

Reaction 40: polyP enhances XI activation by thrombin (5).

A simple reaction for polyP enhancing XI activation by thrombin would be $XI + IIa + polyP \rightarrow XIa + IIa + polyP$. The rate law would be:

$$\frac{d[XIa]}{dt} = k_{1,39}[XI][IIa][polyP]$$

For an estimate of the reaction rate constant for polyP enhancing XI activation, Fig. 1a from Reference shows a XI activation rate of ~1 nM/min when ~5 μM polyP is present. The concentrations used in this figure were $[XI] = 30\,nM$ and $[IIa] = 5\,nM$. Therefore,

$$k_{1,40} \sim \frac{1\frac{nM}{min}}{(30\,nM)(5\,nM)(5\,\mu M)} {\sim} 2.2 \cdot 10^{10}\frac{1}{M^2 s^1}$$

For all polyP reactions, elementary mass action kinetics was assumed. Also, the experiments that the rate constants were based were in bulk solutions, whereas the experiments performed for this paper were in microfluidic devices. To account for this, a factor η was multiplied to each polyP forward rate constant ($k_{1,38}$, $k_{1,39}$, $k_{1,40}$) and simulations were run to match the clotting time in the microfluidic device. η was found to be ~0.4, so the final rate constants used are as appears in Table S1 ($k_{1,38}$ = $4.0 \cdot 10^{5}$ $M^{-1}s^{-1}$, $k_{1,39}$ = $8.0 \cdot 10^{12}$ $M^{-2}s^{-1}$, $k_{1,40}$ = $8.8 \cdot 10^{9}$ $M^{-2}s^{-1}$).

Supplementary Table S2: Chemical species and their initial concentrations used in the simulations for Fig. 1. All initial concentrations were taken from Reference (20).

Species	Initial Concentration (M)
VII	$1 \cdot 10^{-8}$
VIIa	$1 \cdot 10^{-10}$
Xa	0
IIa	0
X	$1.6 \cdot 10^{-7}$
IX	$9 \cdot 10^{-8}$
II	$1.4 \cdot 10^{-6}$
VIII	$7 \cdot 10^{-10}$
VIIIa	0
IXaVIIIa	0
IXaVIIIaX	0
VIIIa1L	0
VIIIa2	0
V	$2 \cdot 10^{-8}$
Va	0
XaVa	0
XaVaII	0

mIIa	0
TFPI	$2.5 \cdot 10^{-9}$
XaTFPI	0
ATIII	$3.4 \cdot 10^{-6}$
XaATIII	0
mIIaATIII	0
IXaATIII	0
IIaATIII	0
XII	$3.4 \cdot 10^{-7}$
XIIa	0
XIIaXII	0
PK0	$4.5 \cdot 10^{-7}$
XIIaPK	0
XIIK	0
K	0
C1inh	$2.5 \cdot 10^{-6}$
XIIaC1inh	0
XIIaATIII	0
XI	$3.1 \cdot 10^{-8}$
XIIIa	0
XIa	0
XIIaXI	0
XIaATIII	0
XIaC1inh	0
a1AT	$4.5 \cdot 10^{-5}$
a2AP	$1 \cdot 10^{-6}$
XIaa1AT	0
XIaa2AP	0
XIaIX	0
IXaX	0
XaVIII	0
VIIaIX	0
VIIaX	0

KInhibited	0
polyP	**

**For the polyP concentration, the same number of polyP molecules ($7.5 \cdot 10^{-9}$ moles) was either i) localized to the surface of the cylinder, or ii) dispersed throughout the volume of the cylinder. This corresponds to a surface polyP concentration of $3 \cdot 10^{-5}$ mol/m^2 in the SI-polyP simulations and a dispersed polyP concentration of $3 \cdot 10^{-2}$ mol/m^3 in the D-polyP simulations.

Supplementary Table S3: Shear rates used in the simulations for Fig. 1b and the resulting thrombin concentration for dispersed and localized polyP.

Shear rate	$[\text{thrombin}]_{SI\text{-}polyP}$ (mol/m^3)	$[\text{thrombin}]_{D\text{-}polyP}$ (mol/m^3)	$[\text{thrombin}]_{SI\text{-}polyP}$ / $[\text{thrombin}]_{D\text{-}polyP}$
1	$1.83 \cdot 10^{-5}$	$2.34 \cdot 10^{-8}$	782.1
4	$1.72 \cdot 10^{-9}$	$1.65 \cdot 10^{-11}$	104.2
12	$4.70 \cdot 10^{-12}$	$1.93 \cdot 10^{-13}$	24.4
24	$8.40 \cdot 10^{-14}$	$2.98 \cdot 10^{-14}$	2.8
60	$1.87 \cdot 10^{-15}$	$1.95 \cdot 10^{-15}$	1.0
120	$2.40 \cdot 10^{-16}$	$2.36 \cdot 10^{-16}$	1.0

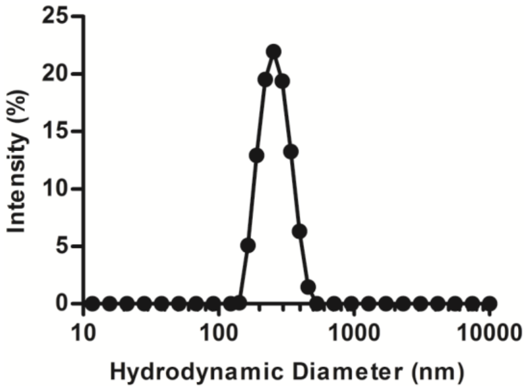

Supplementary Figure S1. Size distribution of NP-polyP$_{160}$. Representative DLS data demonstrating NP-polyP$_{160}$ consisting of particles with an average hydrodynamic diameter of 250 ± 65 nm.

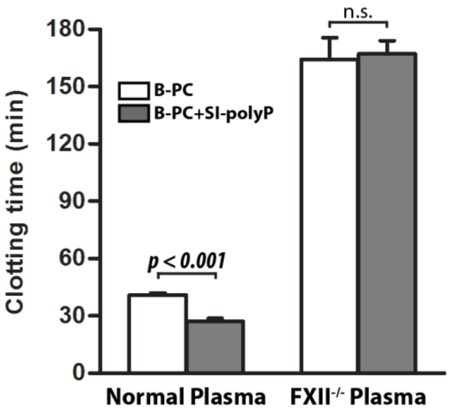

Supplementary Figure S2. polyP facilitates clotting through activation of Factor XII. Clotting times of normal plasma and FXII-deficient plasma at zero-shear with SI-polyP$_{70}$ or without (biotinylated-PC alone). Data indicate mean ± SEM, *n=3.*

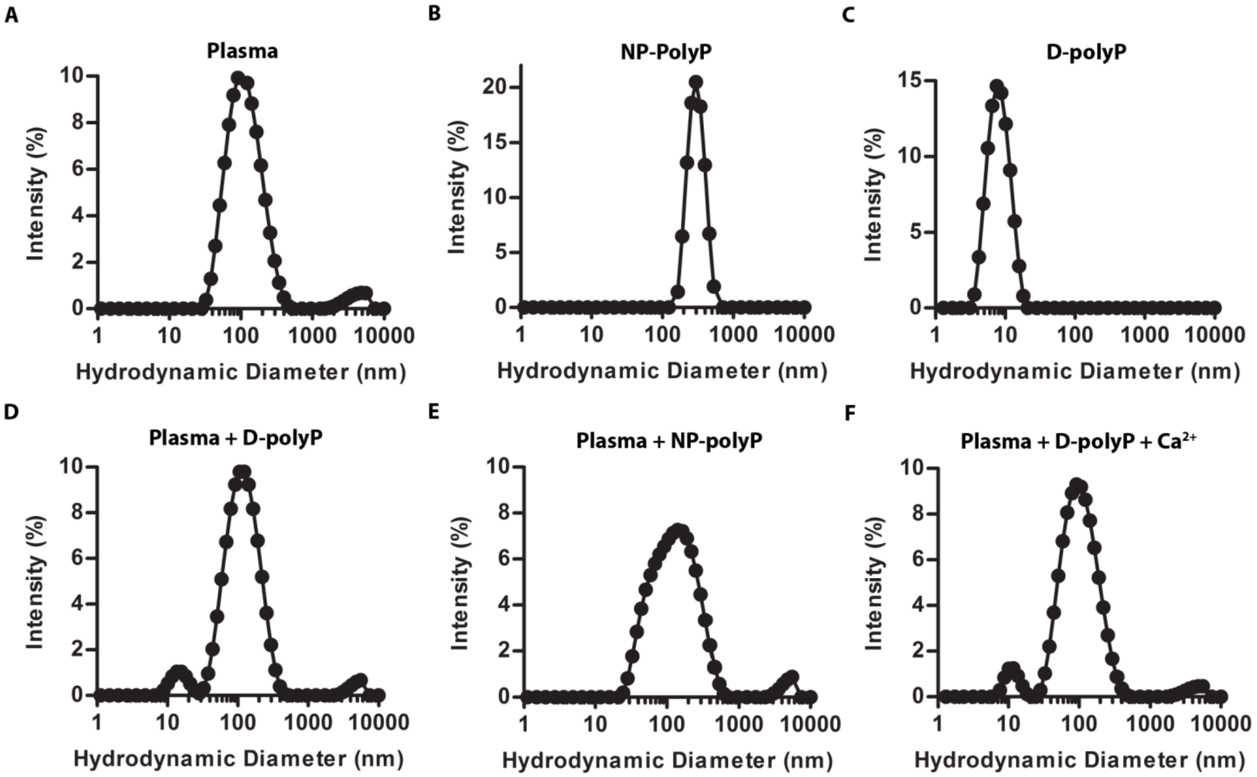

Supplementary Figure S3. DLS size distribution of soluble D-polyP_{160} and NP-polyP_{160} in buffer and plasma. (A) Normal citrated plasma without polyP, which contains background intensity from components normally in plasma. (B-C) Aggregated and soluble polyP_{160} respectively in HEPES buffered saline. (D) Soluble polyP_{160} added to citrated plasma. (E) Preformed NP-polyP_{160} added to citrated

plasma. (F) Soluble polyP$_{160}$ was added to citrated plasma and recalcified; the graph of panel F resembles panel D (D-polyP) rather than panel E (NP-polyP).

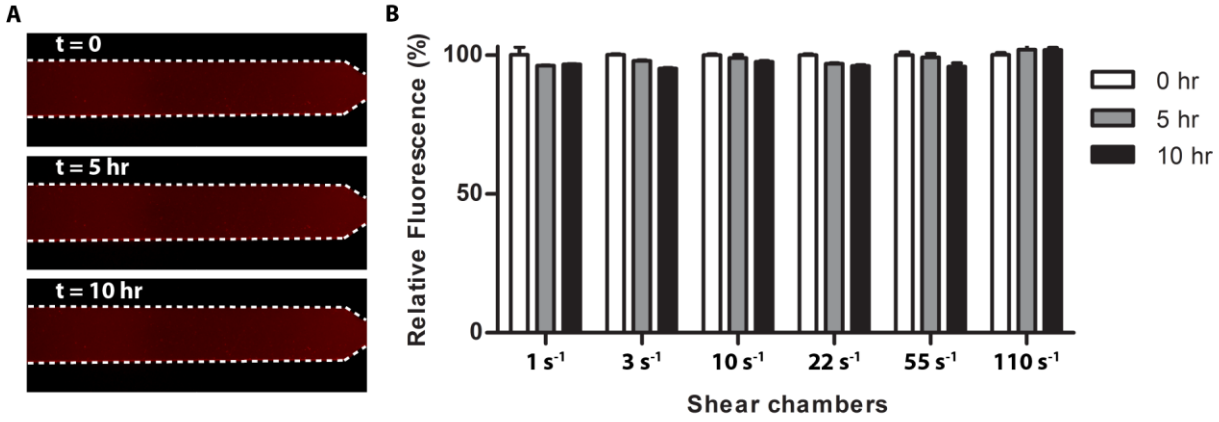

Supplementary Figure S4. PC channel coverage and stability. (A) Representative time lapse images of a channel (white dashed lines) coated with PC/Texas Red DHPE (red) with citrated normal plasma flowing through it. (B) Fluorescence intensities in different regions of each shear chamber. Data indicate mean ± SEM.

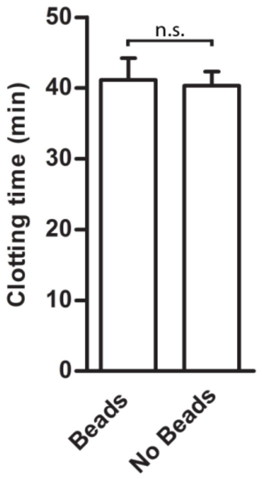

Supplementary Figure S5. Fluorescent beads do not influence clotting. Normal recalcified plasma (with or without beads) was flowed into the device and clotting times at zero-shear were measured by bright-field microscopy. Data indicate mean ± SEM, *n=3.*
